# Implant-Assisted Removable Partial Dentures with Surveyed Crowns: A Narrative Review of Treatment Concepts, Material Considerations, and Clinical Outcomes

**DOI:** 10.3390/dj14090591

**Published:** 2026-09-13

**Authors:** Isaraporn Phusadeekunpaisan, Pimduen Rungsiyakull, Pisaisit Chaijareenont

**Affiliations:** Department of Prosthodontic, Faculty of Dentistry, Chiang Mai University, Chiang Mai 50200, Thailand

**Keywords:** implant-assisted removable partial dentures (IARPDs), implant-surveyed crown removable partial denture (ISCRPD), prosthesis biomechanics, complications

## Abstract

**Background:** Tooth loss remains a major clinical challenge, affecting mastication, speech, facial aesthetics, and oral health-related quality of life. Prosthetic rehabilitation must therefore balance functional demands, biological limitations, patient preferences, maintenance capacity, and cost. Implant-assisted removable partial dentures (IARPDs) have emerged as a practical alternative to conventional removable partial dentures (RPDs) and full-arch fixed implant prostheses, particularly in distal-extension cases where conventional RPDs often demonstrate greater displacement and unfavorable stress distribution. **Objectives:** This narrative review summarizes current concepts in IARPDs treatment, with an emphasis on attachment-retained designs and implant-surveyed crown removable partial dentures (ISCRPDs). **Methods:** A narrative literature review was conducted using PubMed and Scopus. English-language articles related to IARPDs, surveyed crowns, attachment systems, material considerations, biomechanics, clinical outcomes, and complications were identified and narratively synthesized. **Results:** Available evidence suggests that IARPDs provide superior patient-reported outcomes compared with conventional RPDs, largely through improved retention, stability, comfort, and reduced prosthesis movement. Attachment systems offer versatile retention and aesthetic advantages but may require regular maintenance because of component wear and progressive loss of retention. In contrast, surveyed crowns provide a space-efficient and potentially more cost-effective design, with favorable implant survival and prosthetic outcomes reported in retrospective studies. However, complications such as clasp loosening, sore spots, crown dislodgement, and crown or framework fracture remain clinically relevant. Material selection for frameworks and implant crowns further influences aesthetics, durability, hygiene, and long-term maintenance. **Conclusions:** IARPDs treatment should be individualized according to the available prosthetic space, residual ridge anatomy, implant position, oral hygiene ability, aesthetic expectations, financial limitations, and access to follow-up care. Prospective comparative studies are needed.

## 1. Introduction

One of the most significant dental issues for patients is tooth loss, which can be attributed to a multitude of factors, including lifestyle, underlying diseases, history of traumatic events, and dental care [1,2,3]. Tooth loss can have a detrimental impact on a patient’s quality of life, affecting their ability to eat, speak, and maintain an aesthetic appearance [4]. The number of teeth lost can range from a single tooth to the full mouth. Consequently, the treatment will vary according to the type of tooth loss that the patient has.

Treatment decisions are made in collaboration with the patient, with the aim of identifying the most beneficial treatment option for the individual. The treatment options for tooth loss include removable partial dentures, bridges, and dental implants. Several treatment options for edentulism include removable dentures and fixed restoration [5]. Moreover, in the event of the complete loss of all teeth, the available treatment options include a complete denture, an overdenture, and a full fixed prosthesis supported by implants [6,7].

### 1.1. Removable Partial Denture (RPD): Comparison Between Each Types

As previously stated, one of the most prevalent treatments for tooth loss is a removable partial denture (RPD). This can be further classified into two categories: implant-assisted removable partial denture (IARPD) and conventional RPD.

A previous systematic review comparing IARPDs with conventional RPDs assessed patient outcomes using the Oral Health-Related Quality of Life Questionnaire (OHRQoL), the Oral Health Impact Profile (OHIP-49), the Short-Form Health Survey (SF-36), a Visual Analog Scale (VAS), and a patient satisfaction questionnaire. As these instruments are negatively correlated with symptom severity, lower scores indicate better quality of life. The review found that IARPDs provided significantly better outcomes than RPDs showing higher QOL and satisfaction in IARPDs [8].

Building on the improvement in patient-reported quality of life, biomechanical analyses have shown that adding implants to support a removable partial denture markedly reduces both displacement and stress compared with a conventional RPD. In finite element analysis, the conventional RPD model showed greater sagittal displacement toward the natural teeth, with a mean displacement of −190 ± 36 μm. The IARPD with premolar implant placement showed a similar displacement pattern but at an approximately hundred-fold smaller scale, with a mean displacement of −6.30 ± 2.33 μm. Stress distribution also differed among models: the conventional RPD demonstrated the highest framework stress, with a mean von Mises stress of 11.3 ± 14 MPa and a maximum of 200 MPa, mainly concentrated at the direct retainers and connectors. In contrast, IARPDs showed lower stress values, with mean von Mises stresses of 3.04 ± 4.96 MPa in the premolar implant model and 1.81 ± 2.37 MPa in the molar implant model, with stress mainly localized around the matrix [9].

Conventional RPDs exhibited large vertical (tissue-ward) and anterior–posterior displacements, with a mean magnitude of ~730 ± 80 µm, sagittal displacement up to 190 µm, and vertical displacement up to 900 µm; abutment teeth demonstrated combined vertical movement and tipping toward the edentulous span. In contrast, IARPDs with premolar implants (IARPD-PM) showed a similar displacement pattern but at a scale ~100× smaller in the sagittal plane, with a mean magnitude of ~20 ± 6 µm, whereas molar-placed implants (IARPD-MO) achieved the lowest displacement overall (mean 4 ± 0.8 µm; maximum vertical displacement ~5.5 µm) and minimal movement near implant sites. A similar trend was observed for abutment tooth displacement, which was primarily vertical and significantly lower in both IARPDs configurations, especially in IARPD-MO. Regarding stress distribution, conventional RPDs showed the highest von Mises stresses (mean 11.3 ± 14 MPa, max 200 MPa) concentrated in direct retainers, clasps, and connectors, whereas IARPD-PM and IARPD-MO had substantially lower stresses—3.04 ± 4.96 MPa (max 68 MPa) and 1.81 ± 2.37 MPa (max 43 MPa), respectively—with stress mainly localized around the implant matrix. Overall, implant placement, particularly in the molar region, was the most effective in minimizing both displacement and stress in the prosthesis and supporting structures [9].

However, the cost of a full fixed prosthesis represents a significant financial burden, particularly in low to middle income countries. Consequently, full fixed prostheses may not be the most preferable choice for patients experiencing financial deficits. Moreover, full fixed prostheses necessitate considerable surgical expertise, compatibility with the patient’s bone structure, and financial resources. Consequently, IARPD has become a more prominent topic in contemporary dentistry.

### 1.2. Implant-Assisted Removable Partial Dentures (IARPDs)

IARPDs have emerged as a valuable treatment option for patients with tooth loss. First introduced in 1991 by Dr. Ganz [10], IARPDs offer several advantages over traditional RPD. These advantages include enhanced retention and stability of the RPD, reduced ridge resorption, minimized torque on abutment teeth, and improved patient comfort and denture function. Kuroshima classified IARPDs into two types: those using surveyed crowns and those using attachments. IARPDs with attachments can be further classified according to the type of attachment used, including locator, ball, and bar/clip. The diverse positive outcomes of these attachments require less inter-arch space and improved self-alignment [11,12,13,14].

(1) IARPDs with attachment

In general, the use of attachments is a fundamental aspect of IARPDs, significantly enhancing the prosthesis’s retention, stability, and function. These attachments can be further classified according to the type of attachment used, including Locator, ball, and bar–clip systems, each of which offers distinct clinical advantages and limitations.

**Figure 1:**
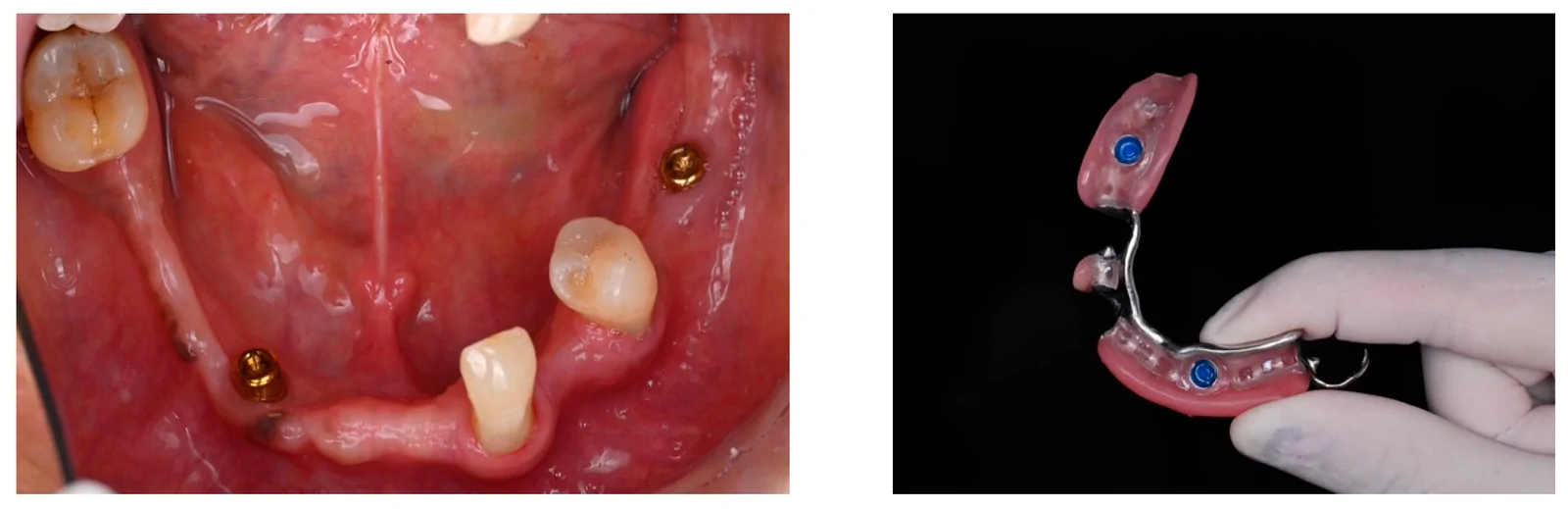
Example of Locator attachment.

Locator attachments are popular for their small size and easy insertion, with a self-aligning feature that helps guide the denture into place as shown in Figure 1. They work especially well when space between the upper and lower teeth is limited, requiring only 7–8 mm [15]. Despite their advantages, Locators are susceptible to wear, resulting in reduced retention over time and often requiring regular replacement of nylon inserts.

Ball attachments, while slightly larger in required space (10 to 12 mm), are valued for their user-friendly design, ease of maintenance, and cost effectiveness. However, like Locators, they too may experience a decline in retention due to long-term component wear [16].

Bar or clip attachments, which require the most vertical space (11 to 15 mm), provide a splinted implant connection that enhances stability and compensates for implant misalignment, making them particularly effective in complex cases. Nonetheless, they pose challenges in terms of higher cost, technical complexity, and difficulty in repair. Therefore, careful selection of the attachment system is essential, guided by anatomical considerations, prosthetic requirements, long-term prognosis, and patient-specific factors to ensure an optimal treatment outcome [11,17,18].

*Indication for IARPD with Attachment*

The clinical indications for implant-assisted removable partial dentures (IARPDs) with attachments, as outlined by Bortolini et al. [19], reflect a comprehensive and practical approach to addressing partially edentulous cases where conventional prostheses may fall short. IARPDs are particularly indicated in Kennedy Class I or II edentulism, when only one or no canine remains, or when the remaining dentition is significantly worn, compromising support and retention.

IARPDs are also suitable for bilateral Kennedy Class III situations with long-span edentulism, as well as for Kennedy Class IV with long anterior edentulous areas. Additionally, patients who decline full fixed or want to combine fixed and removable options often due to cost, surgical burden, or aesthetic concerns can benefit from this approach. IARPDs are especially useful for those who wish to avoid full palatal coverage or visible clasp assemblies, and for individuals with healthy but reduced tissue support. Furthermore, IARPDs offer a solution in cases where existing RPD demonstrates insufficient retention or when intermaxillary relationships preclude the use of fixed partial dentures. IARPDs reduce the need for extensive tooth preparation, minimize biological and financial costs, and can be especially valuable when residual ridge conditions or systemic health issues limit surgical interventions. The use of attachments in IARPDs strategically enhances prosthetic retention while maintaining a simplified biomechanical model, making them a reliable, aesthetic, and cost-effective solution for complex partial edentulism scenarios [8,11,12].

(2) IARPD with surveyed crown

The use of IARPDs with surveyed crown was reported in 1998 [13,20], indicating that it could serve as an alternative to IARPDs. An alternative approach to attachment-retained IARPDs, Tarnow [20] noted that this method has several benefits, including its limited space occupation, lower cost, comparable benefits to fixed prostheses, and superior retention [13,21].

**Figure 2:**
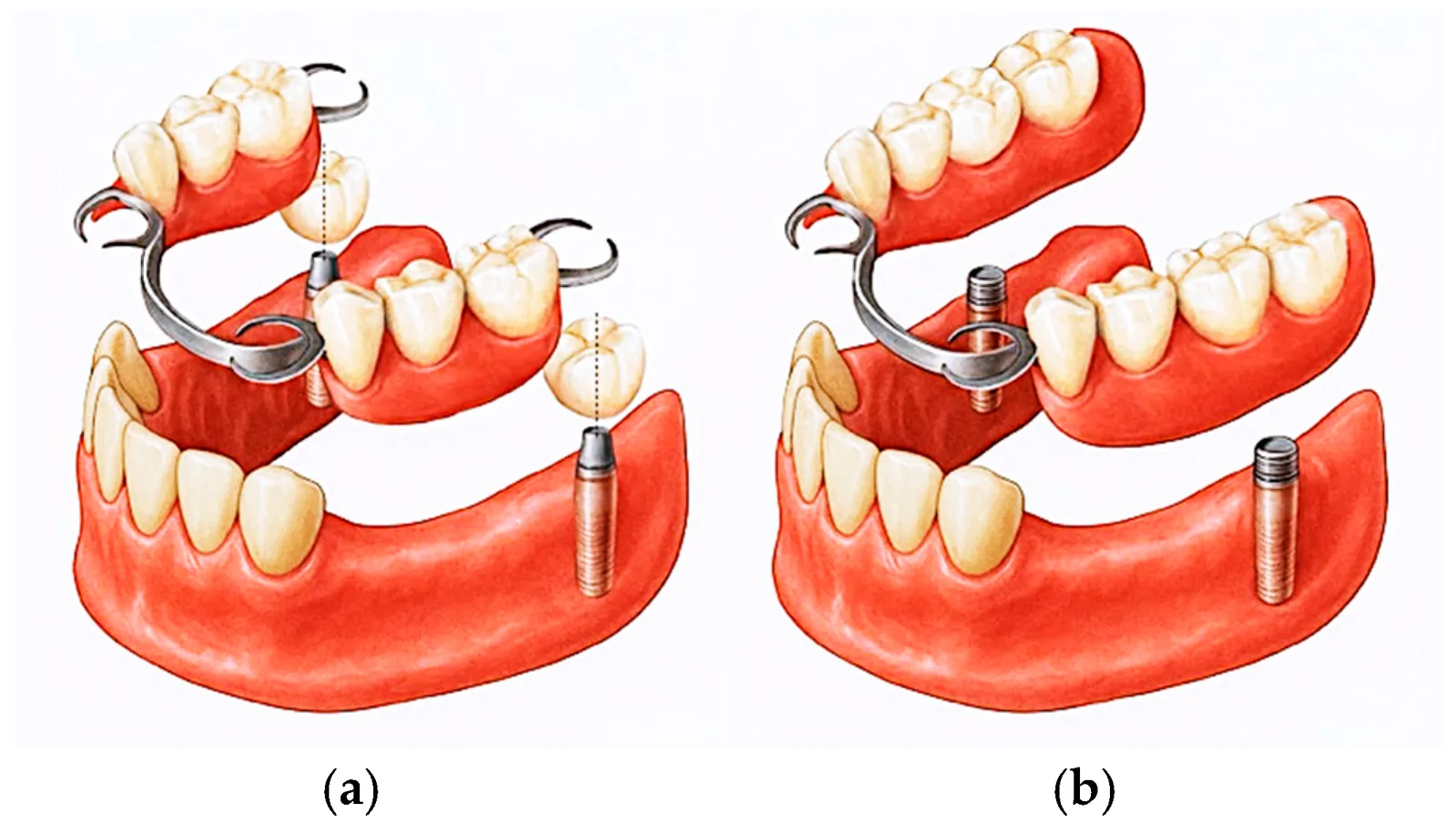
Examples of Implant-assisted removable partial denture (IARPDs) with survey crown (**a**) and IARPD with attachment (**b**).

Figure 2 shows examples of IARPD with attachment and surveyed crown. Implant placement strategies can vary considerably according to the clinical situation and prosthetic design [21,22]. Common configurations found in the literature include tooth-implant-supported, single-implant-supported prostheses, and splinted- or bridged-implant-supported prostheses [13,14]. While these configurations are frequently described in the literature, there is currently no universally accepted classification system for IARPD incorporating surveyed crowns. Despite the lack of formal categorization, these variations have been documented across clinical reports and studies, underscoring the diverse approaches clinicians employ to address distal-extension cases. Understanding these different placement strategies helps clinicians optimize load distribution, enhancing prosthetic stability and longevity.

IARPDs with attachments and IARPDs with surveyed crowns both aim to improve retention and stability over conventional RPDs [8,13,23,24] but differ in application and design. Attachments are favored in Kennedy Class I–II cases with few or worn abutment teeth, long-span Class III or IV situations, or when patients prefer to avoid full fixed options; they offer enhanced aesthetics, avoid full palatal coverage, and require minimal tooth preparation. Surveyed crowns are a space-saving, often lower-cost option that builds implant retained into the crown instead of using separate attachments. They provide retention similar to fixed prostheses and can simplify the prosthesis design, while attachments offer more flexibility for complex ridge or support conditions.

*Indication for IARPDs with Surveyed Crown*

Several potential indications for IARPDs with a surveyed crown have been proposed, including:

1. Patients with limited prosthetic space (less than 6 mm). It is widely acknowledged that each implant requires a distinct volume of space for optimal functionality. The minimum requirement for implants with attachments may vary from 8 to 15 mm, depending on the type of attachment [15,25], However, in IARPDs with a screw-retained surveyed crown, the space requirement is only 4–5 mm. Moreover, no additional surgical procedures are required. Furthermore, in IARPDs with cement-retained surveyed crowns, the space occupation is approximately 6 mm due to the abutment height and the thickness of the crown, which require a certain space. Consequently, IARPDs with surveyed crowns, either screw-retained or cement-retained, require less space than IARPDs with attachments [26].
2. Patients with severe gag reflexes who cannot tolerate irritation from attachment extension. It is well established that the normal population has a certain degree of gag reflex as a defense mechanism against aspiration. However, some individuals exhibit a severe form of gag reflex, affecting up to 15% of the population. This group of people avoided the use of dentures due to the discomfort associated with this condition [27]. The use of IARPDs with a surveyed crown results in a reduction in the maximum extension achieved when an implant is placed in the posterior position to convert a Kennedy Class I situation to Class II. Consequently, the necessity for tissue support is diminished, which ultimately results in a reduction in the patient’s overall discomfort.
3. Patients who lack the time to maintain the attachment, which requires more attention than IARPDs with a surveyed crown. One of the most common complications associated with IARPDs with attachments is the loss of retention in the attachment itself [8,13]. This complication is particularly prevalent in the first year of usage. This complication requires the attention of a specialist, who must diagnose and repair the complication by replacing some or all of the attachment parts. In the case of IARPDs with a surveyed crown, the most commonly observed complication is clasp loosening [21,22,28], which does not require a specialist to perform a specific operation. Consequently, this complication can be managed by a general dentist, thus facilitating patients residing in rural areas or experiencing difficulties in accessing transportation, whether due to geographical constraints or financial limitations. In conclusion, IARPDs with a survey crown may be a preferable option for patients who encounter difficulties in accessing healthcare or who lack sufficient time to meet with a specialist.
4. Patients who prioritize aesthetic aspects due to the similarity of the crown to a natural tooth and gum. With many patients who prefer less denture flange, IARPDs with surveyed crown support this need by fixed restoration when compared with IARPDs with attachments [21]. This concept only applies to certain locations including anterior teeth, and aesthetic zones. Other patients may be satisfied with characteristics of fixed restoration than conventional removable dentures. IARPDs with surveyed crown are composed of both fixed and removable features [19,21,29].
5. Patients with financial limitations, as IARPDs with a surveyed crown are less costly than full fixed prostheses [17].

## 2. Material Used in IARPDs

### 2.1. RPD Framework

- Cobalt-chromium (CoCr): This is the most common material because it is affordable, thin, and widely available. However, it has aesthetic problems as it has a metallic taste [30].
- Titanium alloy (Ti-6Al-4V): While thin and biocompatible, titanium alloy is more difficult to cast and more expensive than CoCr. It is a good option for people allergic to CoCr, but it may have more porosity and slightly less retention compared with cobalt-chromium over time [30,31,32].
- Polyetheretherketone (PEEK): This is a newer material that is non-metallic (a polymer). It is hypoallergenic, polishes well, resists plaque deposition, and is wear-resistant. Additionally, PEEK offers a more aesthetic clasp option as it comes in white, but it is thicker (1.5 mm) compared to the other materials [30,33].

For RPD frameworks, cobalt-chromium (CoCr) remains the most common choice because it is thin, affordable, and widely available, though it has aesthetic drawbacks due to its metallic appearance and taste [30]. Titanium alloy (Ti-6Al-4V) offers similar thinness and excellent biocompatibility, making it suitable for patients allergic to CoCr; however, it is more expensive, harder to cast, and may have greater porosity with slightly reduced retention over time [30,31,32]. PEEK is a non-metallic, hypoallergenic polymer that resists plaque, wears well, and offers a more aesthetic white clasp option, though it is thicker (1.5 mm) and more costly than CoCr [30,33]. The price of the mentioned materials from cheapest to most expensive are CoCr, PEEK, and Ti-6Al-4V.

### 2.2. Crown on Implant (For IARPDs with Surveyed Crown)

- Metal: Full metal crowns (FMCs) are frequently utilized in conjunction with UCLA custom abutments, which are manufactured by waxing on a plastic abutment and then casting metal to create a single unit with the abutment. This approach results in the shortest possible crown height. Gold and other precious metals are the most common materials used for FMCs. However, a disadvantage of FMCs is their limited aesthetics [34,35].
- A porcelain-fused-to-metal (PFM) crown is composed of a metal substructure, typically made of cobalt-chromium or nickel-chromium. One of the primary advantages of PFM crowns is their relatively affordable cost. They are less expensive than all-ceramic crowns. However, there are also some disadvantages to PFM crowns. One of the most notable characteristics of PFM crowns is the visibility of the metal substructure through the porcelain. Additionally, PFM crowns may exhibit chipping or fracturing, and the porcelain may wear away over time [36,37,38].
- All-Ceramic Restorations: All-ceramic restorations offer superior aesthetics and strength compared to traditional metal-based restorations. However, there are two main types of all ceramic restorations: veneered ceramic and monolithic ceramic. Veneered ceramic restorations feature a porcelain layer bonded to a substructure, typically made of zirconia or alumina. This approach provides excellent aesthetics because the porcelain can be customized to match the exact shade and translucency of natural teeth. However, veneered ceramic restorations are more susceptible to chipping, which can compromise their appearance and function. Monolithic ceramic restorations are fabricated from a single block of ceramic material, such as reinforced glass–ceramic or zirconia. This eliminates the potential for chipping, making monolithic restorations more durable than veneered restorations. Additionally, monolithic restorations allow for a completely digital workflow, streamlining the fabrication process [5,36,39].

However, monolithic zirconia restorations have some potential drawbacks. They may be more prone to screw loosening compared to veneered restorations, and there is also a risk of loss of retention and abutment fracture due to their higher stiffness. Furthermore, monolithic reinforced glass–ceramic restorations are more susceptible to core fracture [40,41].

For crowns on implants, full metal crowns (FMCs), often made from gold or precious metals, are durable and allow the shortest crown height when fabricated with UCLA custom abutments, but they lack aesthetic appeal [34,35]. Porcelain-fused-to-metal (PFM) crowns combine a metal substructure with porcelain for acceptable strength and a lower cost than all ceramic options; their downsides include visible metal, porcelain chipping, and gradual wear [36,37,38]. All-ceramic restorations offer superior aesthetics and strength, with veneered ceramics providing highly customizable appearance but a greater risk of porcelain chipping [5,36,39]. Monolithic ceramics, fabricated from a single block of zirconia or reinforced glass–ceramic, eliminate chipping and are highly durable with potential for a full digital workflow; however, their high stiffness can increase screw loosening, risk of retention loss, and abutment fracture, while reinforced glass–ceramic versions may be prone to core fracture [5,36,39,40,41].

## 3. Methods

This narrative review was conducted using literature searches of the PubMed and Scopus databases. Relevant articles were identified using keywords related to implant-assisted removable partial dentures (IARPDs), surveyed crowns, and clinical outcomes. Studies were screened based on predefined eligibility criteria. Articles were included if they evaluated the use of surveyed crowns in IARPDs and reported clinical, functional, prosthodontic, or patient-reported outcomes. Articles not directly related to IARPDs, studies lacking outcome data, conference abstracts, editorials, and non-English publications were excluded. The selected literature was reviewed and synthesized narratively to summarize the current evidence regarding the clinical performance and outcomes of IARPDs utilizing surveyed crowns.

## 4. Result

### 4.1. Clinical Outcome of IARPDs with Surveyed Crown

The clinical outcomes of IARPDs with surveyed crowns are primarily informed by a series of retrospective clinical studies conducted predominantly in South Korea, which may introduce regional and methodological bias (Table 1). Most of these investigations are retrospective case series without control groups, limiting the ability to make robust comparative evaluations, particularly between IARPDs with surveyed crowns and those utilizing alternative retention systems such as attachments. Despite these limitations, the available literature has reported on a diverse range of outcome measures including implant survival rates, marginal bone loss (MBL), prosthetic complications, aesthetic satisfaction, and implant biomechanics.

For instance, Bae et al. found that implant-surveyed crown removable partial dentures (ISCRPDs) demonstrated significantly less marginal bone loss (1.44 ± 0.57 mm) compared to implant overdentures (IODs), although plaque and calculus accumulation were more common in the ISCRPD group [17]. Soo-Hyun Kang et al. reported a high survival rate for surveyed crowns (95.1%) and noted favorable long-term outcomes with ISCRPDs in terms of retention and function [42]. Similarly, You-Kyoung Oh et al. observed no implant failures and acceptable probing depth (3.4 ± 0.1 mm), but documented minor prosthetic complications such as clasp and rest fractures [43].

Soo-Yeon Yoo and colleagues conducted multiple studies comparing ISCRPDs with IODs in both mandibular and maxillary edentulism. Their findings suggest that ISCRPDs had higher implant survival rates (e.g., 97.3% vs. 70.4% in the maxilla) and less bone loss, while IODs were associated with more attachment-related maintenance complications. Moreover, function and patient satisfaction were consistently favorable with ISCRPDs, especially in the mandible, although some abutment tooth failures were noted [21,22,29]. Tae-Wook Jung et al. further confirmed high implant survival (96.9%) and low MBL (0.11 mm) with ISCRPDs, particularly in Kennedy Class I cases [44].

Biomechanical modeling studies by Ju-Won et al. [45] and Eduardo Piza Pellizzer et al. [46] examined stress distribution in various IARPDs designs. Ju-Won et al. demonstrated that implant-tissue-supported RPDs exhibited higher stress and displacement compared to figured- or tooth-supported RPDs, emphasizing the importance of implant positioning. Pellizzer’s study found that combinations using ERA and O-ring attachments produced more displacement and increased stress on the supporting structures, with the ERA-supported model providing the most favorable stress distribution [45,46].

Lastly, Yi et al. [28] evaluated ISCRPD success in Kennedy Class I and II cases using three posterior implant positions. They found the highest success rates (83.3%) when implants were placed adjacent to abutment teeth without altering the dental arch (Type 1a), compared to more distal placements (62.6–73.2%).

### 4.2. Complication Reported in IARPD with Surveyed Crown

The complications reported from the clinical use of IARPDs incorporating surveyed crowns are multifaceted and span across prosthetic, implant, crown, and soft tissue domains. As detailed in Table 2, retention loss particularly in the form of clasp loosening emerged as a frequently observed complication. Notably, clasp loosening was the predominant issue in several studies, including Yoo et al., which reported an incidence as high as 69.23%, and Yi et al., which recorded 41.9%. Yoo et al. (2021) and Yoo et al. (2022) also documented significant rates of clasp loosening at 44.44% and 31.81%, respectively, indicating this as a persistent mechanical challenge in IARPDs framework-related complications [21,22,28,29].

Implant-related complications such as screw loosening were less frequent, observed in only a few studies, including 6.25% in Soo-Hyun Kang et al. [42] and 20% in Tae-Wook Jung et al. [44]. Regarding denture deformation, Bae et al. reported a 100% rate of denture base fracture in their sample, underscoring the vulnerability of the prosthetic base in the early iterations of IARPDs [17]. Additionally, Yi et al. noted fracture rates of 8.1% in both the denture base and artificial teeth, and 4.8% in RPD rest components [28]. Fractures of the RPD clasp were also highlighted, with Oh et al. documenting a relatively high incidence (33.33%), further illustrating the mechanical stresses imposed on distal-extension frameworks [43].

Crown-related complications such as dislodgement and fracture were also prevalent. Dislodgement of the crown was seen in 25% of cases in both Kang et al. and Bae et al., and was also reported by Yoo et al. with a rate of 23.08%. Crown fracture, though less frequent, occurred in 16.67% of cases in both Oh et al. and Kang et al., and in 8.1% in the cohort reported by Yi et al. [17,21,28,42,43].

Tissue-related complications especially sore spots were also reported with concerning frequency. Yoo et al. observed a 22.73% rate of sore spots, the highest among the studies, followed by Yi et al. at [28,29] who reported 24.2%. Other soft tissue problems included loss of osseointegration and marginal bone loss (MBL). Kang et al. recorded a 12.5% rate of osseointegration loss, while Yoo et al. [22,29,42] noted MBL in 7.4% and 9.1% of patients, respectively. In contrast, peri-implantitis, a biologic complication of great clinical significance, showed notable variability in reporting. For example, Yoo et al. [21] specified an 11% prevalence of peri-implantitis in the maxilla, and Yi et al. reported 21 cases without specifying the arch. Yoo et al. [29] was the only study to disaggregate data by arch, reporting a 3% rate in the mandible and no cases in the maxilla [21,29,30].

Other complications, including fracture of opposing teeth and tooth loss, were less consistently reported. However, some studies noted these events under the category of “Others.” For example, Kang et al. [42] and Yoo et al. [29] reported rates of 25% and 22.73%, respectively, for complications classified as “Other,” a category that may include biologic or prosthetic issues not otherwise specified. Yoo et al. [22] also documented a 14.44% rate of sore spots, suggesting that patient comfort remains an important clinical consideration. In terms of overall complication counts, Yi et al. reported the highest number of complications with 62 total events, followed by Yoo et al. [28,29] with 22 events and Kang et al. [42] with 16.

In summary, while the complication profiles vary between studies possibly due to differences in sample size, follow-up duration, and implant positioning strategies, certain trends are evident. Clasp loosening, sore spots, and crown-related complications appear to be the most prevalent across the literature. These findings underscore the importance of meticulous planning, careful prosthetic design, and ongoing maintenance in the long-term success of IARPD with surveyed crowns.

## 5. Discussion

Complications associated with attachment-retained IARPDs include retention loss, attachment wear, marginal bone loss, peri-implant inflammation, and prosthesis maintenance requirements [21,22,29]. Among attachment-retained designs, Locator systems are particularly susceptible to retention loss due to wear of the nylon inserts during repeated functional loading, often necessitating periodic replacement [17]. In contrast, implant-surveyed crown-retained IARPDs rely primarily on conventional removable partial denture components, such as rests, guide planes, and clasps, which can often be adjusted or repaired without the need for specialized attachment components. As a result, complications such as clasp loosening are generally manageable by general dental practitioners, potentially facilitating long-term maintenance, particularly in areas with limited access to specialist care [21,22,28,29,42].

Marginal bone resorption represents another important consideration in the long-term success of IARPDs. Several studies have reported favorable peri-implant bone preservation in surveyed crown-retained designs compared with overdenture-type attachments [21,22,28,42]. Although the exact mechanism remains incompletely understood, the distribution of occlusal forces across both implants and remaining natural teeth may contribute to more favorable biomechanical loading conditions [47]. Supporting this concept, finite element studies have demonstrated that implant assistance can reduce stress concentrations on abutment teeth and supporting tissues, while prosthesis design continues to influence force distribution [48]. Collectively, these findings suggest that the favorable clinical outcomes observed with implant-surveyed crowns may be partly attributable to improved load distribution and reduced biomechanical stress on supporting structures.

From a clinical perspective, implant-surveyed crowns may be particularly advantageous for patients with limited restorative space, high aesthetic demands, or situations in which attachment maintenance may be impractical [20]. In the anterior region, surveyed crowns can reduce the visibility of metal components while maintaining favorable retention, support, and stability [21,22]. Furthermore, their compatibility with conventional removable partial denture principles may simplify future maintenance and repair procedures compared with attachment-retained alternatives.

Despite these advantages, surveyed crown-retained IARPDs are not without limitations. The fabrication of implant-supported surveyed crowns requires precise laboratory procedures and advanced clinical skills, which may increase treatment complexity and overall cost compared with conventional RPDs. Furthermore, in cases of significant alveolar bone loss, achieving favorable implant positioning for surveyed crown fabrication may be challenging and could compromise prosthetic outcomes. Surveyed crown-retained IARPDs may also create plaque-retentive areas [43] that are more difficult for patients to clean than conventional RPD, potentially increasing the risk of plaque accumulation, calculus formation, and peri-implant inflammation if oral hygiene is inadequate. Therefore, careful patient selection, thorough preoperative planning, and a structured maintenance program are essential to minimize biological and technical complications.

It is also important to distinguish between implant-retained and implant-supported RPD designs. In implant-retained or implant-assisted RPDs, the implant should not be considered the sole source of support; rather, support should continue to be provided by the denture-bearing mucosa, residual ridge, and conventional RPD components, while the implant contributes to retention, stability, and load sharing [13,19,23,24]. If excessive functional load is transferred to the implant, potential complications may include screw loosening, marginal bone loss, peri-implant inflammation, or mechanical overload of the prosthetic components [17,21,22,28,42,44]. Therefore, implant position, occlusal scheme, prosthesis fit, and adequate tissue support should be carefully planned to reduce the risk of overloading the implant and to support favorable long-term outcomes [14,28,45,46,47].

Overall, the available evidence suggests that implant-surveyed crown-retained IARPDs represent a predictable treatment option within the spectrum of implant-assisted removable prostheses, offering favorable clinical outcomes, satisfactory aesthetics, and manageable maintenance requirements [17,21,22,28,29,42,43,44]. Among the included retrospective studies, Kang et al. reported a 95.1% implant survival rate for surveyed crown-retained IARPDs [42]. Yoo et al. reported higher implant survival rates for ISCRPDs than IODs in mandibular edentulism (98.3% vs. 83.1% at 8 years) and maxillary edentulism (97.3% vs. 70.4%) cohorts, respectively [21,22]. Yi et al. further reported that treatment success varied according to implant position, with higher success when implants were placed adjacent to natural teeth (83.3%) than in more distal positions (62.6–73.2%) [28]. However, the current evidence base remains limited by the predominance of retrospective studies, small sample sizes, and heterogeneity in prosthetic designs and outcome measures. Consequently, direct comparisons between surveyed crown-retained designs and attachment-retained IARPDs should be interpreted with caution.

Despite the promising clinical findings reported for implant-surveyed crown-retained IARPDs, the certainty of the available evidence remains low. Nearly all clinical data included in this review were derived from retrospective case series, predominantly conducted in a single country and, in several studies, from largely overlapping research groups. The absence of randomized controlled trials or well-designed prospective comparative studies limits the ability to establish causality or to directly compare surveyed crown-retained IARPDs with attachment-retained designs or conventional RPDs. Moreover, the available studies may be affected by selection bias, reporting bias, differences in patient characteristics, implant positioning, prosthetic design, follow-up duration, and maintenance protocols. These limitations reduce the external validity and generalizability of the findings to broader clinical populations and different healthcare settings. Therefore, although the current evidence suggests that implant-surveyed crown-retained IARPDs may be a clinically feasible and useful treatment option, the conclusions should be interpreted with caution.

Future research should focus on prospective, multicenter studies with standardized outcome measures to better evaluate long-term implant survival, peri-implant bone changes, prosthetic maintenance requirements, and patient-reported outcomes. Comparative studies evaluating implant-surveyed crowns against Locator-, ball-, and bar-retained IARPDs would be particularly valuable for establishing evidence-based treatment selection criteria. Additionally, further investigation into the influence of clasp design, implant position, restorative materials, and digital workflows may help optimize treatment planning and improve the long-term success of implant-assisted removable partial dentures.

## 6. Conclusions

IARPDs are a practical option for partially edentulous patients, especially in distal-extension cases where conventional RPDs may provide limited retention and stability. Attachment-retained designs are useful when additional retention and aesthetics are required, but they may need regular maintenance due to attachment wear. In contrast, implant-surveyed crown RPDs may be advantageous for patients with limited prosthetic space, financial constraints, aesthetic concerns, or reduced access to specialist care. Common complications include clasp loosening, sore spots, crown dislodgement or fracture, plaque accumulation, and peri-implant tissue responses. Material selection should be individualized, as CoCr, titanium, PEEK, metal, PFM, and ceramic crowns each present different trade-offs in cost, aesthetics, strength, space requirement, and maintenance.

## Figures and Tables

**Table 1:** Clinical outcomes of implant-assisted removable partial dentures (IARPDs) with surveyed crowns.

Author	Year	Country	Type of Study	Objective	Outcome	Types of IARPDs	Conclusion
Bae et al.	2017	Korea	Clinical Retrospective Study	To compare clinical findings of implant-supported removable partial dentures	Implant cumulative, Survival rate, Marginal bone resorption, Probing depth, Peri-implant inflammation, Bleeding, Plaque, Calculus, Complications	ISCRPD and IOD	ISCRPD exhibited significantly lower marginal bone loss (1.44 ± 0.57 mm). However, calculus buildup was significantly more prevalent in the ISCRPD group. Other outcomes were not significant
Ju-Won et al.	2017	Korea		To investigate the biomechanical interactions between implant crowns, bone, RPD, and IARPDs.	Von Mises stress, Displacement of the denture abutment tooth, Displacement of the implant system	Tooth-supported RPD (TB), Implant-supported RPD (IB), Tooth-tissue-supported RPD (TT), Implant-tissue-supported RPD (IT)	Model IT exhibited twice the von Mises stress of model IB, while model TT matched model TB. Maximum displacement was higher in TB and TT compared to IB and IT, with the highest in TT and lowest in IB.
Soo-Hyun Kang et al.	2020	Korea	Clinical Retrospective Study	To explore survival rate of IARPDs	Survival of the implant: Location, RPD classification, Opposing dentition, Splinting, Implant diameter	ISCRPD and IOD	Survival rate of surveyed crown: 95.1%, overdenture: 88.2%.
You-Kyoung Oh et al.	2021	Korea	Clinical Retrospective Study	To analyze the performance and complication of ISCRPD	Implant cumulative survival rate, Marginal bone resorption, Probing depth, Peri-implant inflammation, Bleeding, plaque, Calculus, Complications	ISCRPD	No implant failure 2 clasp fractures, 1 rest fracture, 1 decementation, 1 fracture of porcelain on an implant-surveyed prosthesis, Mean bone loss of 0.77 ± 0.63 mm & probing depth of 3.4 ± 0.1 mm.
Soo-Yeon Yoo et al.	2021	Korea	Clinical Retrospective Study	To assess survival rate and MBL between ISCRPD and IOD in Mandibular Edentulism.	Survival rate, MBL PROMs on functional Aesthetic satisfaction Complications	ISCRPD and IOD	ISCRPDs showed higher implant survival by Kaplan–Meier survival curves (98.3% vs. 83.1% at 8 years) and lower bone loss compared to IODs. Both improved patient function and aesthetics, but IC-RPDs had more clasp issues while IODs had more attachment problems.
Soo-Yeon Yoo et al.	2021	Korea	Clinical Retrospective Study	To assess survival rate and MBL between ISCRPD and IOD in Maxillary Edentulism.	Survival rate, MBL PROMs on functional Aesthetic satisfaction Complications	ISCRPD and IOD	IC-RPDs outperform IODs in both implant survival rate (97.3% vs. 70.4%) and bone preservation (1.12 vs. 3.31 mm). While both improve satisfaction, IODs experience more complications.
Soo-Yeon Yoo et al.	2022	Korea	Clinical Retrospective Study	To assess survival rate and MBL in ISCRPD	Survival rate, MBL PROMs on functional Aesthetic satisfaction Complications	ISCRPD	All implants survived treatment and showed minimal bone loss. Both function and aesthetics improved significantly with ISCRPD, with greater improvement in function for implants in the mandible. IC-RPDs rarely had complications, but some abutment teeth failed. Notably, abutment tooth bone loss was lower than implant bone loss.
Tae-Wook Jung et al.	2023	Korea	Clinical Retrospective Study	To assess the clinical outcomes of posterior implants with ISCRPD	Clinical outcomes, Mechanical problems (implant length, crown-to-implant (C/I) ratio), MBL	ISCRPD	High implant survival (96.9%) and success (90.6%) rates were observed. Mean bone loss was low (0.11 mm), with Kennedy class II showing more loss.
Yuseung Yi et al.	2023	Korea		To assess ISCRPD success in Kennedy Class I & II based on 3 implant placement strategies in back jaw areas (Type 1a, Type 1b and Type 2)	Success (implant-related complications. Kaplan–Meier curves and the multivariable Cox regression model)	- Anterior implants adjacent to the abutment tooth without modification of arch (1a) - Anterior implants apart from the abutment teeth in distal-extension area (2a) - Posterior implants in the distal-extension area to modify from Kennedy Class I or II to Class III (2)	IARPD success varied by implant position: higher near natural teeth (1a) (83.3%) than further away (2a,2) (62.6–73.2%). Overall treatment success rate was 66.7%.
Eduardo Piza Pellizzer et al.	2010	Brazil		To assess the biomechanics of a lower implant-supported distal-extension removable partial denture with varying retention systems (Five models were tested: A (canine + DERPD), B (canine + implant + healing abutment + DERPD), C (canine + implant + ERA attachment + DERPD), D (canine + implant + O-ring attachment + DERPD), E (canine + implant-supported prosthesis + DERPD)	Stress value Displacement	Hemimandible (class I) with - Canine support (A) - Canine and implant with a healing abutment support (B) - Canine and implant with an ERA attachment support (C) - Canine and implant with an O-ring attachment support (D) - Canine and implant-supported prosthesis (E)	Model C showed the best stress distribution in the supporting tissues, indicating it caused less strain on the surrounding bone. Models B, D, and E displayed similar stress patterns; however, model E resulted in higher displacement and overload, suggesting it may put too much stress on the supporting tissues.

**Table 2:** Complications reported in IARPD with surveyed crown.

Author	Year	Retention Loss	Implant	Denture Fx/Deformation				Crown		Tissue			Others Complication	Total Number of Survey Crown on Implant and RPD Complications	Percent of Peri-Implantitis (Mn/Mx/NA)
		Clasp Loosening	Screw Loosening	Fx of Denture Base	Fx of Artificial Tooth	Fx of RPD Rest	Fx of RPD Clasp	Dislodgement Crown	Fx of Crown	Loss of Osseointegration	MBL	Sore Spot			
Bae et al.	2017	0	0	6(100%)									0	6	21.70%(NA)
Soo-Hyun Kang et al.	2020	1(6.25%)	1(6.25%)		1(6.25%)	1(6.25%)		4(25%)	1(6.25%)	2(12.5%)	1(6.25%)		4(25%)	16	
You-Kyoung Oh et al.	2021					1(16.67%)	2(33.33%)	1(16.67%)	1(16.67%)		1(16.67%)		0	6	27%(NA)
Soo-Yeon Yoo et al.	2021	12(44.44%)	0	0	0	0	1(3.7%)	0	0		2(7.4%)	12(14.44%)	0	25	
Soo-Yeon Yoo et al.	2021	9(69.23%)	0	0	0	0	0	3(23.08%)	0		0	1(7.69%)	0	13	11%(Mx)
Soo-Yeon Yoo et al.	2022	7(31.81%)	0		0	1(4.55%)	0	2(9.1%)	0		2(9.1%)	5(22.73%)	5(22.73%)	22	0(Mx) 3%(Mn)
Tae-Wook Jung et al.	2023		1(20%)							1(20%)			3(60%)	5	4.54%
Yuseung Yi et al.	2023	26(41.9%)		5(8.1%)	2(3.2%)	5(8.1%)	3(4.8%)		5(8.1%)	1(1.6%)			15(24.2%)	62	21(NA)

Gray-shaded cells indicate data not reported in the corresponding study. NA, minimum or maximum value not specified.

## Data Availability

No new data were created or analyzed in this study. Data sharing is not applicable to this article.

## Abbreviations

IARPD	Implant-assisted removable partial denture
RPD	Removable partial dentures
ISCRPD	Implant-surveyed crown removable partial dentures
PEEK	Polyetheretherketone
PFM	Porcelain-fused-to-metal
OHRQoL	Oral Health-Related Quality of Life Questionnaire
OHIP	Oral Health Impact Profile
SF	Short-Form Health Survey
VAS	Visual Analog Scale
IOD	Implant over denture
MBL	Marginal bone loss
